# Pancreatic enzyme supplementation after gastrectomy for gastric cancer: a randomized controlled trial

**DOI:** 10.1007/s10120-017-0757-y

**Published:** 2017-08-14

**Authors:** Marco Catarci, Manuele Berlanda, Giovanni Battista Grassi, Francesco Masedu, Stefano Guadagni

**Affiliations:** 1 General and Oncologic Surgery, Department of Oncology, San Filippo Neri Hospital, Rome, Italy; 2 Department of Applied Clinical Sciences and Biotechnology, University of L’Aquila, L’Aquila, Italy; 3 Direttore UOC Chirurgia Generale, Ospedale “C. e G. Mazzoni”, AV5-ASUR Marche, Via degli Iris, 63100 Ascoli Piceno, Italy

**Keywords:** Stomach neoplasms, Gastrectomy, Exocrine pancreatic insufficiency

## Abstract

**Background:**

Gastrectomy for gastric cancer is a significant cause of secondary exocrine pancreatic insufficiency. Pancreatic enzyme replacement therapy may influence nutritional status and quality of life after gastrectomy, but the pertinent clinical research to date remains controversial. A randomized controlled trial to test this hypothesis was carried out.

**Methods:**

After gastrectomy, 43 patients with gastric cancer were randomly assigned to a normal diet (Normal-d; *n* = 21) or to a pancreatic enzyme supplementation diet (PES-d; *n* = 22) and were followed up during a 12-month period, assessing nutritional status and quality of life through body mass index (BMI), instant nutritional assessment (INA) class status, serum pre-albumin (SPA) values, and GastroiIntestinal Quality of Life Index (GIQLI).

**Results:**

BMI was not significantly influenced by the type of diet; INA class status was significantly improved in the PES-d arm, particularly during the first 3 months after gastrectomy; SPA levels increased in both arms at 6 months after gastrectomy, reaching significantly higher values in the PES-d arm at 12 months. GIQLI was not significantly influenced by the type of diet throughout the follow-up period; however, this index significantly improved in the PES-d arm between the first and third month after gastrectomy.

**Conclusions:**

PES-d improves nutritional status and quality of life after gastrectomy for gastric cancer, particularly within 3 months from the operation. A larger, multicenter trial is necessary to address the potential influence of several confounding variables such as disease stage and adjuvant treatments.

## Introduction

Despite a clear decline in incidence during the past decades, gastric cancer still represents the second most common cause of death from cancer worldwide [1, 2]. Overall survival rates for gastric cancer increased from 4% in 1971–1975 to approximately 30% in 1973–2010 [3, 4]; consequently, evaluation of nutritional disturbances after gastrectomy presents a developing issue [5–10]. Actually, inadequate pancreatic enzyme release, resulting from reduced secretive response to endogenous stimuli or reduced pancreatic enzyme activation after gastrectomy because of bacterial overgrowth, has been commonly reported [5, 7, 8, 11]. This “secondary” pancreatic insufficiency is generally considered different from the primary type ensuing from the loss of pancreatic parenchyma (surgery, necrosis, fibrosis, neoplasms) or dysfunction of enzyme-secreting acinar or ductal cells [12], because direct pancreatic stimulation tests generally give a normal response after administration of secretagogues [13].

Extent of gastric resection (total/subtotal gastrectomy) and the type of reconstruction (duodenal passage of food preservation or bypass) can influence the degree of pancreatic insufficiency, which is generally more severe after total gastrectomy and after duodenal bypass reconstructive techniques [12–15]. Moreover, it seems that pancreatic insufficiency after gastrectomy decreases in the long-term period, but without returning to the preoperative status, demonstrating that the intact pancreatic parenchyma alone is not sufficient to compensate for reduced gastric functions [16].

Studies on pancreatic enzyme supplementation (PES-d) after gastrectomy available to date have given discordant results. The first one, performed in 1968, demonstrated a significant reduction of fecal fats with PES after Billroth II subtotal gastrectomy [17]. The first randomized trial, nearly 20 years later, failed to demonstrate reduction of steatorrhea with PES-d [18], but the same authors published contradictory results only 1 year later [16]. More recently, the same group published a randomized controlled trial on a larger series (52 cases) that demonstrated a significant benefit of PES-d on overall quality of life without any effect on steatorrhea, bowel movements, and caloric intake [19]. The limitations of the study design were that the observation time was very short (14 days), in addition to the use of a lipid-rich diet (48%) that is unrealistic and hardly reproducible in clinical practice.

We therefore decided to investigate the role of PES-d after gastrectomy for gastric cancer by means of a randomized controlled trial.

## Methods

This study is a single-center randomized controlled trial intended to compare the effect of long-term PES-d over normal diet (Normal-d) after total gastrectomy (TG) or distal sub-total gastrectomy (DSG). The following hypothesis has been tested: four parameters related to nutritional status and quality of life after long-term PES-d diet or Normal-d are different.

The four parameters, the endpoints of the study, were body mass index (BMI, kg/m^2^), Instant Nutritional Assessment (INA) [20] [allocating patients into four ordinal nutritional classes (1 = best; 4 = worst) according to lymphocyte count and serum albumin levels], serum pre-albumin (SPA) levels, and quality of life measured with the Gastrointestinal Quality of Life Index (GIQLI) [21].

Concerning study populations and eligibility criteria, all consecutive patients aged over 18 years and referred to total or distal subtotal gastrectomy for gastric cancer were screened for inclusion in the trial, which started in November 2012. Inclusion criteria included informed consent. Patients referred to proximal gastrectomy or wedge resection were excluded, as they share more than half of the residual stomach and preservation of duodenal transit of food; in particular, duodenal transit stimulates cholecystokinin production by duodenal and jejunal cells with pancreatic hormonal stimulation of exocrine digestive enzymes [22]. Other exclusion criteria were metastatic gastric carcinoma, secondary tumor, and active chronic gastrointestinal disease. Patient screening included a standard preoperative evaluation of the anesthesiology class (ASA) and a baseline assessment based on BMI, INA, SPA levels, and GIQLI.

The sample size was determined from the study hypothesis to require at least 20 patients per arm to detect an expected 20% difference in the BMI change, according to a type I error of 5% and a power of 80%. To reduce selection bias, randomization was conducted using a block-randomization schedule designed to balance PES-d and Normal-d in the two arms, given the type of gastrectomy.

Concerning trial timeline, a total of 50 patients with gastric cancer per year were expected to be referred to the trial institution; a recruitment rate of 70% was expected. The recruitment of 40 patients was planned to be complete within 15 months. The time interval from first patient to last patient out was 16 months.

All patients were submitted to open surgery through a laparotomic approach (upper midline or bilateral subcostal based on tumor location and surgeon’s preference). Indication for DSG or TG and extent of lymph node dissection have been previously described [23, 24]. Reconstruction of digestive tract continuity after DSG consisted of a Roux-en-Y single-layer hand-sewn gastrojejunostomy and a Roux-en-Y stapled (25-mm circular stapler) esophagojejunostomy after TG. After DSG, the rationale of Roux-en-Y (RY) reconstruction is based on a lower incidence of both heartburn symptoms and endoscopic grade of gastritis, in comparison to Billroth II, as recently confirmed by a randomized controlled trial [25]. Cholecystectomy was performed for prophylactic intention and for gallstones, assuming that cholecystectomy increases cholecystokinin production by duodenal and jejunal cells only in the presence of duodenal transit [22]. Excision of other structures and organs was performed when necessary. To minimize performance bias, all gastrectomies were performed by the same team in an elective setting under a strict perioperative protocol concerning antibiotic and antithrombotic prophylaxis, blood transfusions, surgical drainage placement/removal, and oral intake of food after the operation. Postoperative complications were assessed and graded according to the Clavien–Dindo classification [26, 27].

**Fig. 1:**
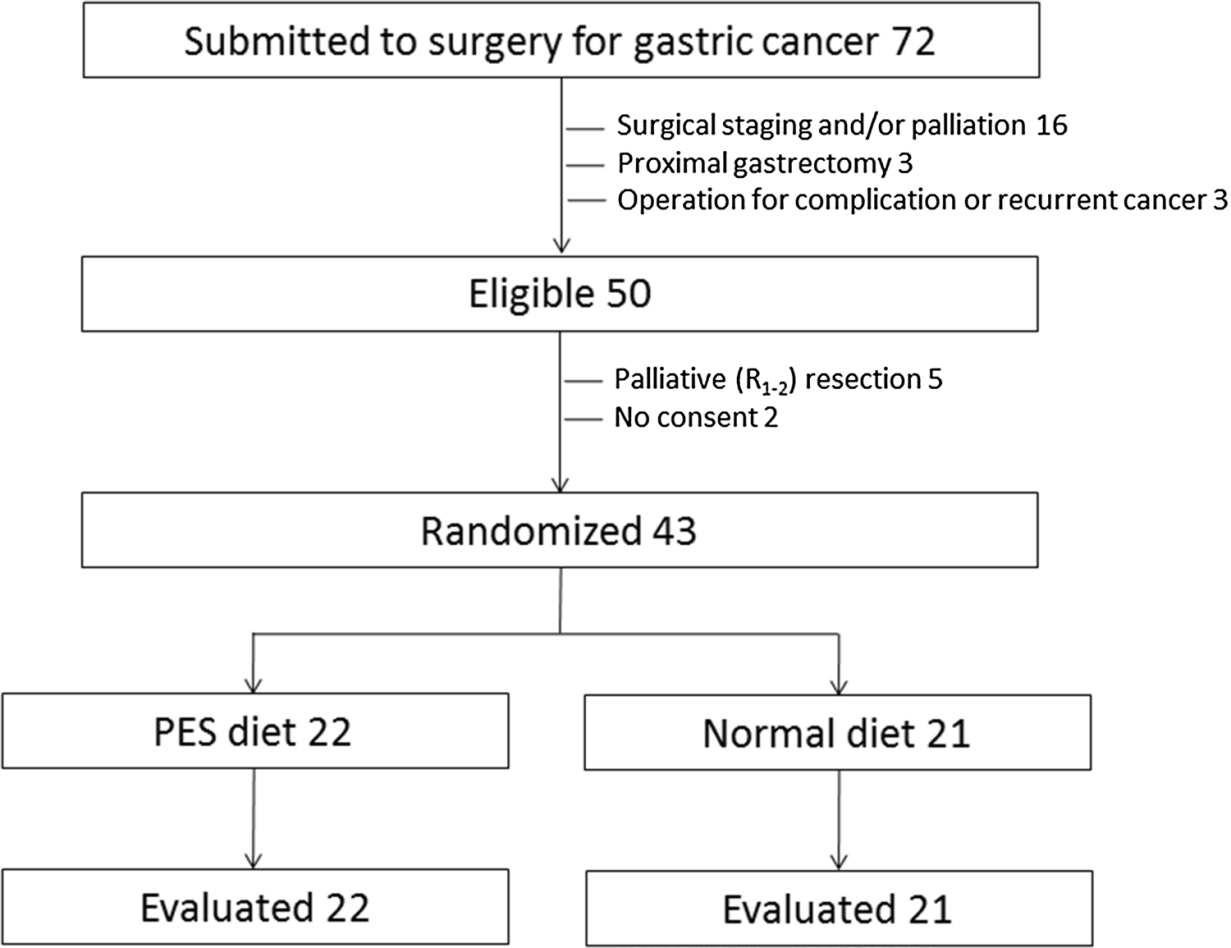
Enrollment flowchart. *PES* pancreatic enzyme supplementation

After 20 months from the start of the trial (June 2014), 72 patients with gastric cancer had been surgically treated in our unit. Twenty-two cases were excluded preoperatively by surgical staging and/or palliation in 16, proximal gastrectomy in 3, and operation for complication or recurrent cancer in 3 cases. Overall, 50 patients were included in the study. Postoperatively, 7 cases were excluded because of unplanned *R*_1–2_ resection in 5 cases and consent denial in 2 cases; the remaining 43 cases were randomized (Fig. 1). There were 19 women and 24 men, aged 66.1 ± 12.4 years (mean ± SD). Patient, tumor, and treatment characteristics in the two randomization arms are shown in Table 1.

**Table 1:** Patient, tumor, and treatment variables in the two randomization arms (*PES* pancreatic enzyme supplementation)

	Normal diet		PES diet		Exact Fisher *p* value
	No.	%	No.	%	
Gender					
Female	13	61.9	11	50.0	0.543
Male	8	38.1	11	50.0	
ASA class					
I–II	15	71.4	12	54.5	0.347
III–IV	6	28.6	10	45.5	
Gastrectomy					
Subtotal distal gastrectomy	15	71.4	17	77.3	0.736
Total gastrectomy	6	28.6	5	22.7	
Lymph node dissection					
D_1_	12	57.1	9	40.9	0.366
D_2_	9	42.9	13	59.1	
pT-stage					
Not advanced gastric cancer (pT1/pT2)	10	47.6	16	72.7	0.124
Advanced gastric cancer (pT3/pT4)	11	52.4	6	27.3	
Cholecystectomy					
Yes	3	14.3	6	27.3	0.457
No	18	79.1	16	72.7	
Postoperative adjuvant therapies					
Yes	5	23.8	7	31.8	0.736
No	16	76.2	15	68.2	

Ten to 18 days after their operation, two types of diet (Normal-d and PES-d) were assigned to the patients based on randomization. Normal-d was based on food consumption five or six times per day, with a relatively high level of carbohydrates and a normal level of both fat and medium-chain triglycerides. PES-d was the same as the normal diet, with the addition of oral administration of capsules of CREON 10,000 U. Ph. Eur. (distributed in Italy by Abbott S.r.l., cat. AIC 029018), each containing 150 mg pancrelipase microgranules (amylase 8,000 U. Ph. Eur.—lipase 10,000 U. Ph. Eur.—protease 600 U. Ph. Eur.), derived from pig pancreatic parenchyma. The dose was one capsule at breakfast, two capsules at lunch, and two capsules at dinner, with the instruction to open the capsules and add the microgranules to semisolid acid food (pH <5.5) with limited chewing or to acid liquids (pH <5.5), according to the specific indications of the manufacturer for gastrectomized patients.

All patients submitted to DSG received proton pump inhibitors because pancrelipase microgranules are irreversibly deactivated by gastric stump acid [28]. All patients were followed up at an ambulatory checkup scheduled at 1, 3, 6, and 12 months postoperatively, which included the medical history, a clinical examination, and an assessment of the four endpoints. No patient was lost to follow-up. The endpoints were graded and calculated by blinded assessors based on ambulatory records.

### Statistical analysis

Relative to the study design, the statistical analysis had to manage a longitudinal factorial design with a 12-month follow-up.

Endpoints were characterized by three continuous outcomes and one ordinal score, respectively: BMI, SPA, GIQLI, and INA (ordinal). The outcomes were assessed with linear mixed models with interactions using an unstructured covariance matrix. In particular, the analysis allowed estimation of the two diets and the main effects of gastrectomy. Furthermore, time changes in the outcome variables were estimated using planned time contrasts. Model fitting was calculated using a Wald *χ* ^2^.

The INA status analysis was addressed similarly using a linear mixed regression assuming a latent linear construct for the ordinal responses. As the INA status is an ordinal index stemming from continuous variables, the latent linear construct is justified.

Statistical significance level was set at 5%; model fitting and contrasts were Sidak adjusted for multiple tests. Statistical analysis was carried out using STATA statistical software (release 14; StataCorp, College Station, TX, USA).

## Results

All postoperative adverse events are reported in Table 2. One patient in the Normal-d arm was submitted to reoperation on postoperative day 2 to control ongoing abdominal cavity bleeding; the following postoperative period was uneventful, and oral intake was resumed on postoperative day 8. Four further major adverse events occurred in the postoperative period: one duodenal stump dehiscence after DSG in the PES-d arm, conservatively treated with oral intake resumed on day 11; two cases with infected abdominal collections, one in each study arm, both treated by means of percutaneous drainage and i.v. antibiotics with oral intake resumed on day 11 and day 12, respectively; and one patient in the Normal-d arm experienced myocardial ischemia on postoperative day 3, conservatively treated in the postoperative period without any impact on oral intake of food; this patient was submitted to percutaneous myocardial revascularization 3 months later. We recorded no perioperative death and no evidence of recurrent or persistent disease during the follow-up period.

**Table 2:** Grading of complications in the two randomization arms

	Normal diet		PES diet		Exact Fisher *p* value
	No.	%	No.	%	
Grade I					
Wound infection	2	9.5	3	13.6	0.999
Other	2	9.5	1	4.5	
Grade II					
Myocardial ischemia	1	4.7	–	–	
Duodenal stump leakage	–	–	1	4.5	
Grade IIIa					
Percutaneous drainage of abscess	1	4.7	1	4.5	
Grade IIIb					
Reoperation for bleeding	1	4.7	–	–	
Total	7	33.3	7	31.8	

All but one of the patients randomized to PES-d tolerated oral administration of pancreatic enzymes during the study period; minimal side effects were reported in two cases (nausea, early satiety). PES-d was suspended at 6 months because of intolerable bloating in one patient who was included in the PES-d arm evaluation on an intention-to-treat basis.

### BMI

**Fig. 2:**
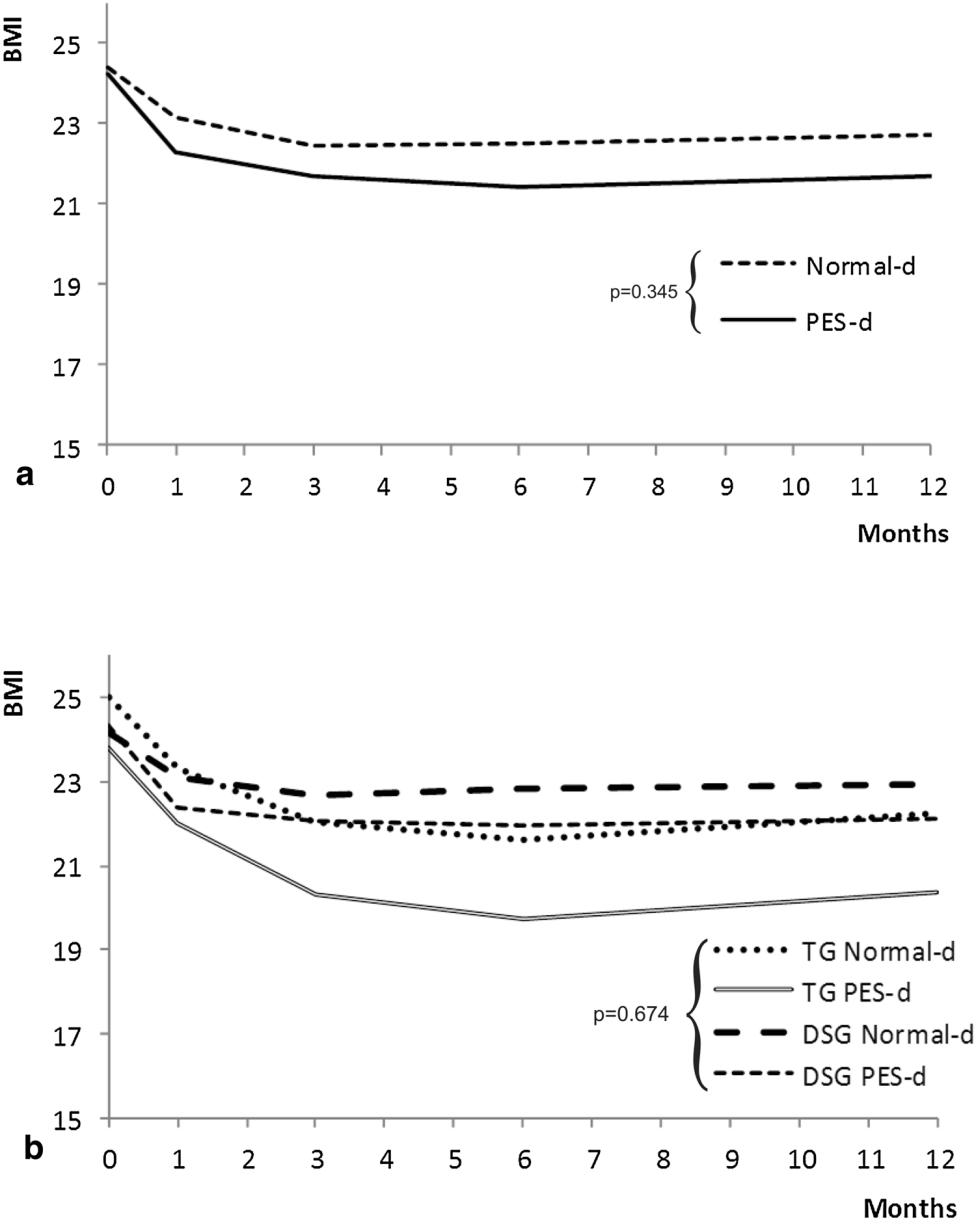
Time trend analysis of body mass index (BMI) according to randomization arm (**a**) and randomization arm and type of surgery (**b**). *PES* pancreatic enzyme supplementation, *TG* total gastrectomy, *DSG* distal subtotal gastrectomy, *d* diet

The linear mixed model was fitted to the BMI data (Wald $\chi_{df = 17}^{2}$ = 130; *p* < 0.001). Gastrectomy caused significant decrease of BMI with time (Wald $\chi_{df = 4}^{2}$ = 115.66; *p* < 0.001). The interaction between the type of surgery (DSG vs. TG) and time was statistically significant (Wald $\chi_{df = 4}^{2}$ = 14.39; *p* < 0.01). Diet type (Wald $\chi_{df = 4}^{2}$ = 4.48; *p* = 0.34; Fig. 2a) and the other variables, i.e., pT-stage, cholecystectomy, and postoperative adjuvant therapies, had no statistically significant impact on this factor.

Based on time trend, maximal BMI decrease (−3.23 ± 0.57 kg/m^2^) was predicted at 6 months. Type of gastrectomy was statistically significant (DSG more than TG) in determining BMI recovery from the third month until the end of the follow-up (*p* < 0.02). The difference between DSG and TG became apparent after the third month (Fig. 2b).

### INA

**Fig. 3:**
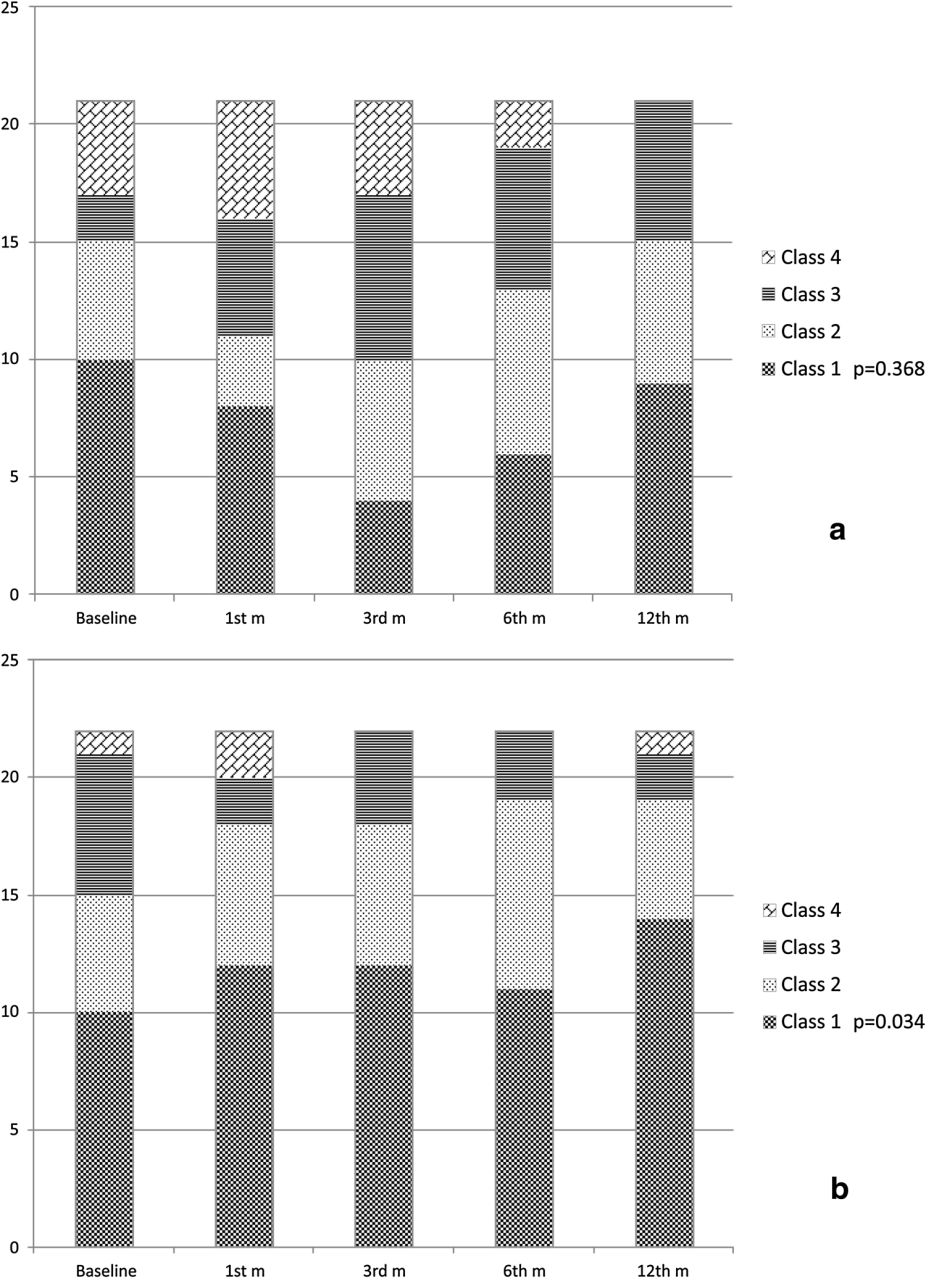
Time trend analysis of INA class status according to the randomization arm: normal diet (**a**); PES diet (**b**)

The linear mixed model was fitted to the INA data (Wald $\chi_{df = 17}^{2}$ = 27.46; *p* < 0.03). The PES-d significantly improved the INA class status in patients submitted to gastrectomy with time (Wald $\chi_{df = 4}^{2}$ = 10.44; *p* = 0.03), particularly during the first 3 months (Fig. 3). This characteristic loses statistics significance when subdividing the patients in relationship to the extent of gastrectomy (Wald $\chi_{df = 4}^{2}$ = 1.35; *p* < 0.85). The other variables had no statistically significant impact on INA class status.

### SPA

**Fig. 4:**
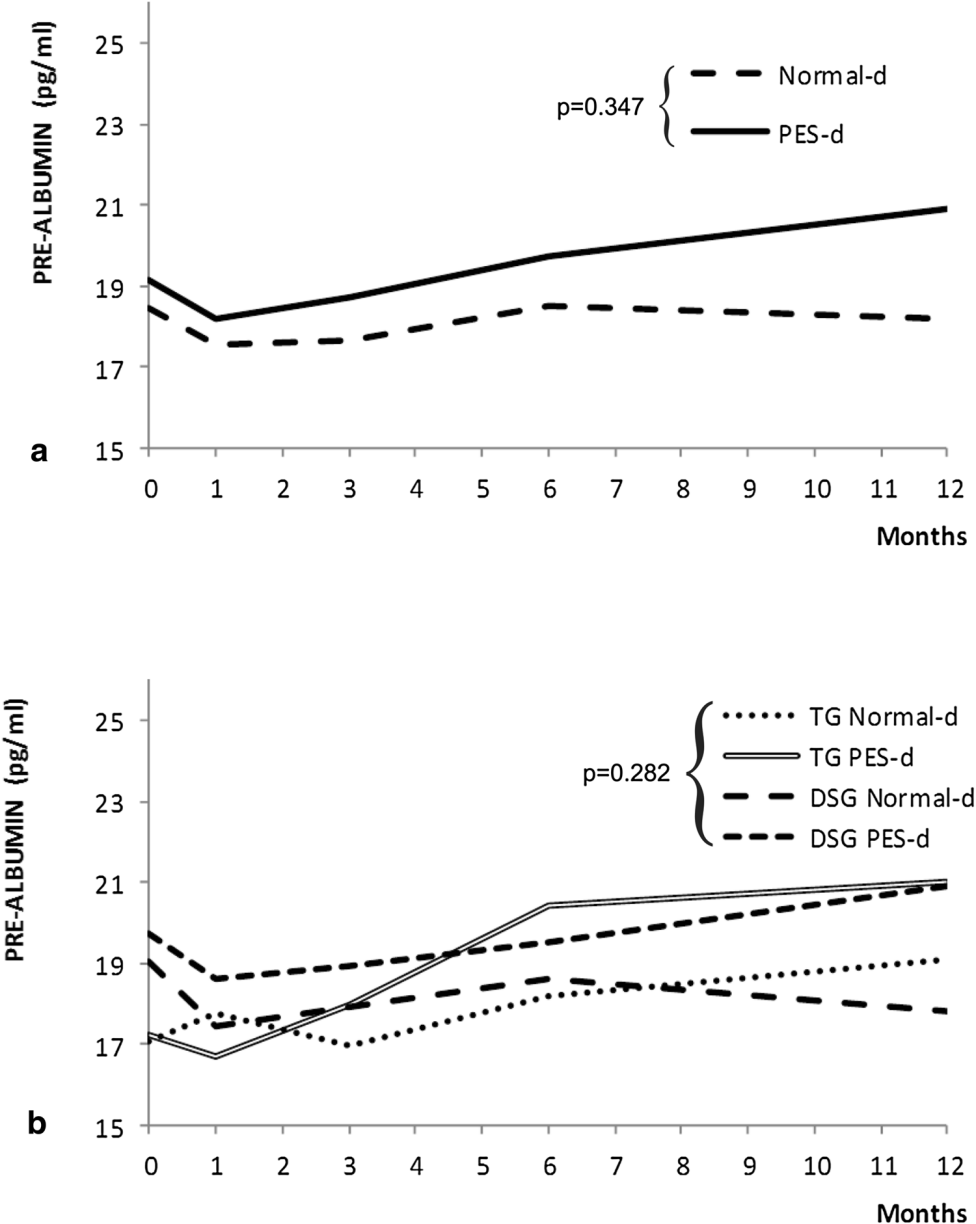
Time trend analysis of serum pre-albumin according to randomization arm (**a**) and randomization arm and type of surgery (**b**). *PES* pancreatic enzyme supplementation, *TG* total gastrectomy, *DSG* distal subtotal gastrectomy, *d* diet

The linear mixed model was fitted to SPA data (Wald $\chi_{df = 17}^{2}$ = 30.57; *p* < 0.001). After gastrectomy, the SPA values did not change statistically with time (Wald $\chi_{df = 4}^{2}$ = 6.64; *p* < 0.16). Type of diet (Fig. 4a; Wald $\chi_{df = 4}^{2}$ = 4.44; *p* = 0.35) and the other variables had no statistically significant impact. After the sixth month, the average SPA values showed a statistically significant increase (*p* < 0.04), independently from the type of diet. Only at the twelfth month did the PES-d have a statistically significant impact in comparison to the normal diet in both groups of gastrectomy (Fig. 4b; *p* < 0.05).

### GIQLI

The linear mixed model was fitted to these data (Wald $\chi_{df = 17}^{2}$ = 84.22; *p* < 0.001). After gastrectomy, the average GIQLI score significantly decreased with time (Wald $\chi_{df = 4}^{2}$ = 72.31; *p* < 0.001).

**Fig. 5:**
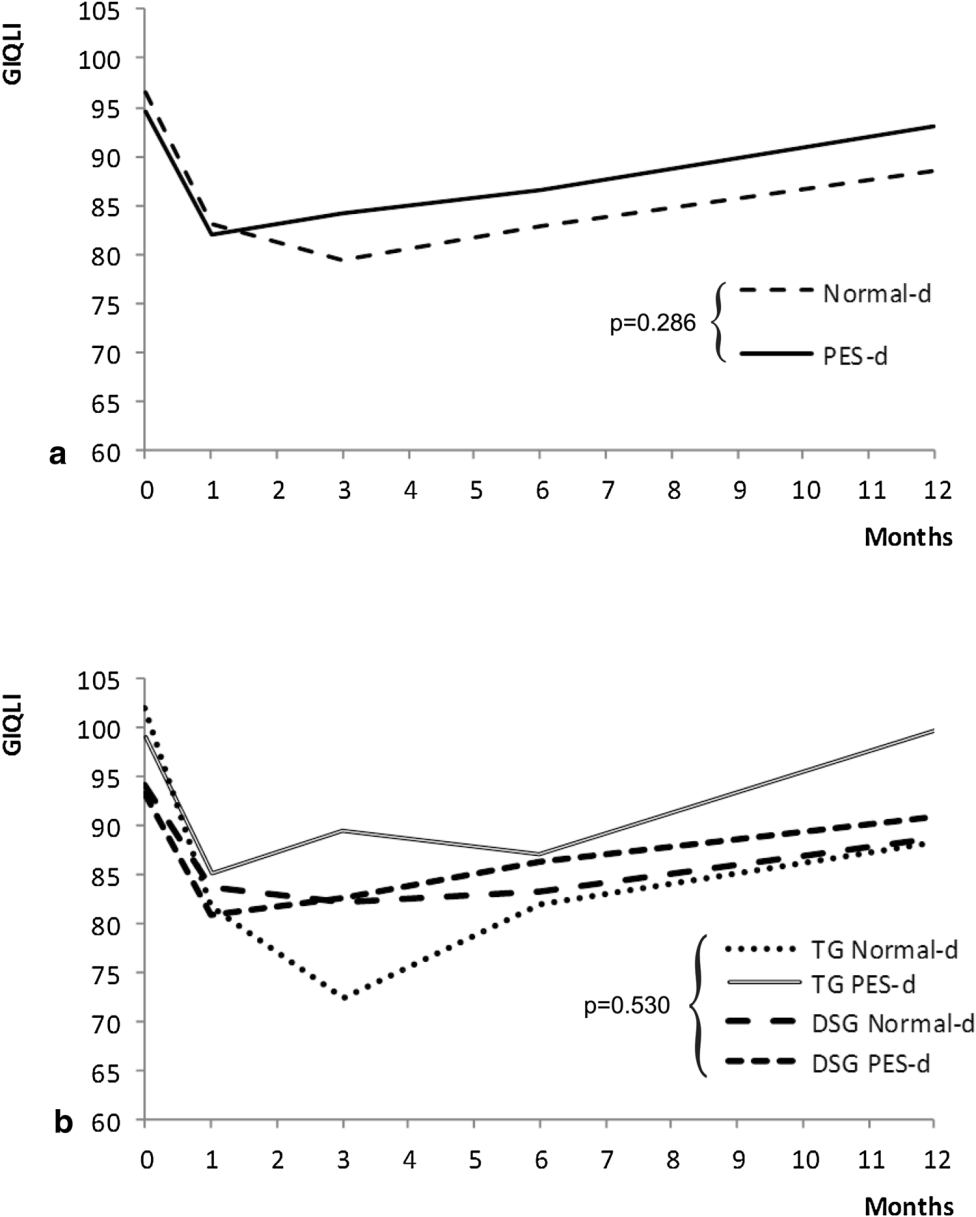
Time trend analysis of GIQLI score according to randomization arm (**a**) and randomization arm and type of surgery (**b**). *PES* pancreatic enzyme supplementation, *TG* total gastrectomy, *DSG* distal subtotal gastrectomy, *d* diet

Type of surgery (Wald $\chi_{df = 4}^{2}$ = 4.85; *p* < 0.30), type of diet (Wald $\chi_{df = 4}^{2}$ = 5.05; *p* = 0.28), and the other variables had no statistically significant impact on this phenomenon over the whole study period; between the first and the third month, the average GIQLI score significantly began to improve in patients receiving PES-d (*p* < 0.05; Fig. 5a).

Concerning the differences between patients submitted to DSG or TG (Fig. 5b), maximal decrease of the average GIQLI score compared to the baseline was at the third month (−14.55 ± 0.31, *p* < 0.001) for DSG with Normal-d, at the first month (−11.3 ± 2.9, *p* < 0.001) for DSG with PES-d, at the third month (−23.3 ± 4.2, *p* < 0.001) for TG with Normal-d, and at the first month (−17.2 ± 4.4, *p* < 0.001) for TG with PES-d; thereafter, a slow increase toward baseline values continued in all subgroups until the twelfth month.

## Discussion

Although pancreatic exocrine insufficiency has been clearly demonstrated after gastrectomy [5, 7, 8, 11], the key question remains whether pancreatic enzyme supplements may improve the clinical outcome of gastric cancer patients, as the available evidence to date remains controversial. A recent position statement of the Italian Association for the Study of the Pancreas [28] clearly states that after gastrectomy for cancer it is mandatory to resort to pancreatic enzyme replacement therapy, orally ingested, during meals, in addition to dietary changes; on the other hand, other sources underline that the effect of high-dose pancreatic enzyme supplementation on symptoms and steatorrhea after gastrectomy is marginal and does not justify its routine use [29]. A randomized controlled trial on this issue was therefore warranted, as recommended in a recent review by Straatman et al. [30]. The first finding of this study is that BMI is significantly reduced up to the sixth month after surgery, slowly recovering thereafter, with significant differences in favor of DSG over TG (Fig. 2) that had been described earlier [31]. This finding is consistent with historical series of surgery for peptic ulcer [10], confirmed also after gastrectomy for gastric cancer [32], in which persistent body weight loss over the sixth month after surgery is strongly indicative of disease recurrence [33]. PES-d showed no significant influence on this phenomenon, casting doubts about its clinical relevance. Although many different index and score systems are used for nutritional assessment in adults [34], INA remains a simple and fast index of nutritional status (being based on serum albumin levels and on lymphocyte count) that is still used in clinical practice [20, 35, 36], and it was significantly improved in the PES-d arm of the study, particularly during the first 3 months after surgery (Fig. 3). Conversely, serum pre-albumin levels failed to show a trend during the whole follow-up period, with a significant improvement after the sixth month that reached a significant difference in favor of the PES-d arm at the twelfth month (Fig. 4). Serum albumin has a half-life at 18–20 days and is the parameter most extensively used for nutritional assessment. Low serum albumin (<2.2 g/dl) is a marker of a negative catabolic state and a predictor of poor outcome [37]. Surgical stress, other acute stresses, hepatic disease, and renal disease decrease serum albumin levels. SPA has a shorter half-life at 2–3 days, responds quickly to the onset of malnutrition, and rises rapidly with adequate protein intake; however, SPA levels can be altered in the acute-phase response by acute or chronic inflammation. In general, inflammatory cytokines reduce the level of pre-albumin synthesis by the liver, and it can also be reduced with renal and hepatic disease. Therefore, SPA is less helpful for assessing overall nutritional status. GIQLI is a 36-item questionnaire querying symptoms, physical status, emotions, social dysfunction, and effects of medical treatment [21], representing a widely accepted tool to investigate quality of life after gastrointestinal surgery [38–41]. In this study, the average GIQLI score began to improve significantly in patients receiving PES-d (*p* < 0.05; Fig. 5a) between the first and the third month, confirming the same trend observed in INA class status.

This study has some qualifying aspects: sample selection, homogeneity of surgical techniques and perioperative management, balanced study design, and blind assessment of outcomes. On the other hand, it has one main limitation: the disproportion of cancer stages in the two arms (Table 1). Postoperative adjuvant treatments for gastric cancer, either chemotherapy or chemoradiation, are associated with an incidence of gastrointestinal toxicity up to 80%, graded as severe in 20% of cases [42, 43]. The direct consequences of this toxicity on food intake, nutritional status, and quality of life after gastrectomy are self-evident. Moreover, stage and adjuvant treatments are not reliably predictable based on pre- and intraoperative findings; consequently, they cannot be randomized. To minimize these selection biases, a sample size of approximately 520 patients has been estimated for a new multicenter randomized controlled trial. The estimate has exploited the BMI effect size stemming from this pilot study, given the type of gastrectomy, stage, and adjuvant therapy predictors, and it has been corrected for a 10% proportion of patients lost to follow-up, setting type I error at 5% and power at 80%.

In conclusion, PES-d seems to improve some nutritional aspects and quality of life after gastrectomy, particularly between the first and third months after surgery. The long-term effects of PES-d may be confounded by the toxicity related to adjuvant treatments, and a larger, multicenter trial is necessary to overcome this potential bias.
